# Surgical innovation revisited: A historical narrative of the minimally invasive “Agarwal sliding‐clip renorrhaphy” technique for partial nephrectomy and its application to an Australian cohort

**DOI:** 10.1002/bco2.78

**Published:** 2021-03-12

**Authors:** A. W. Silagy, R. Young, B. D. Kelly, F. Reeves, M. Furrer, A. J. Costello, B. J. Challacombe, N. M. Corcoran, J. Kearsley, P. Dundee, D. K. Agarwal

**Affiliations:** ^1^ Department of Urology Royal Melbourne Hospital Melbourne VIC Australia; ^2^ Department of Urology Austin Health Melbourne VIC Australia; ^3^ Department of Urology Western Health Melbourne VIC Australia; ^4^ Department of Urology Inselspital, Bern University Hospital Bern Switzerland; ^5^ Epworth Healthcare Melbourne VIC Australia; ^6^ Guy’s and St Thomas’ NHS Foundation Trust London UK

**Keywords:** partial nephrectomy, renorrhaphy, surgical techniques, surgical history, complications

## Abstract

**Objective:**

To evaluate local clinical outcomes of sliding clip renorrhaphy, from inception to current utilization for open, laparoscopic, and robotically assisted partial nephrectomy.

**Methods:**

We reviewed prospectively maintained databases of three surgeons performing partial nephrectomies with the sliding‐clip technique at teaching hospitals between 2005 and 2019. Baseline characteristics, operative parameters, including surgical approach, RENAL Nephrometry Score, and post‐operative outcomes, including Clavien‐Dindo classification of complications, were recorded for 76 consecutive cases. We compared perioperative and 90‐day events with patient and tumor characteristics, stratified by operative approach and case complexity, using Wilcoxon rank‐sum test for continuous variables and the Chi‐squared or Fisher's exact test, for binary and categorical variables, respectively.

**Results:**

Open surgery (n = 15) reduced ischemia time and operative time, but increased hospital admission time. Pre‐ and post‐operative estimated glomerular filtration rates did not change significantly by operative approach. Older patients (*P* = .007) and open surgery (*P* = .003) were associated with a higher rate of complications (any‐grade). Six grade ≥3 complications occurred: these were associated with higher RENAL Nephrometry Score (*P* = .016) and higher pathological tumor stage (*P* = .045). Limits include smaller case volumes which incorporate the learning curve cases; therefore, these data are most applicable to lower volume teaching hospitals.

**Conclusion:**

The sliding‐clip technique for partial nephrectomy was first described by Agarwal et al and has low complication rates, acceptable operative time, and preserves renal function across open and minimally invasive surgeries. This series encompasses the initial learning curve with developing the technique through to present‐day emergence as a routine standard of practice.

## 1 INTRODUCTION

In 1870, Gustav Simon performed the first partial nephrectomy.^1^ For the next century, the procedure was confined to obscurity, largely reserved for patients with solitary kidneys, compromised renal function, and bilateral renal masses. This was due to concerns regarding local recurrence from multifocal malignant renal tumors and morbidity associated with intraoperative and delayed hemorrhage.^2^ The advent of computed tomography (CT) increased the incidence of asymptomatic small renal masses, aided pre‐operative planning, and increased the utilization of the partial nephrectomy.^3^

The only randomized control trial comparing partial and radical nephrectomy between 1992 to 2003 demonstrated that partial nephrectomy had improved long term renal function, reducing the incidence of stage 3a and 3b chronic kidney disease; however, for patients with renal cell carcinoma (RCC), there was no difference in local recurrence, cancer‐specific survival or overall survival.^4^, ^5^ Notably, severe hemorrhages and reoperations were significantly higher in the partial nephrectomy arm.^6^ Since this trial, surgical techniques have evolved, including the utilization of minimally invasive surgery (MIS), first laparoscopy, and later robotically assisted surgery, improving the effectiveness and speed of achieving hemostasis thereby reducing operative time and ischemia time.

In 2007, Agarwal et al described the sliding‐clip renorrhaphy technique.^7^ The principles of sliding‐clip renorrhaphy involve fixing the entry and exit points of suture material in the renal parenchyma with Weck Hem‐o‐lok® clips (Teleflex Medical, Research Triangle Park, NC, USA) that slide down the sutures without slipping back. The technique prevents the cheese‐wire effect (when parenchyma is lacerated as sutures are placed under tension), maintains tension on the suture material to ensure sustained parenchymal compression and hemostasis, and negates the need for slower intracorporeal knot tying. An initial clip is slid down the outer layer suture with a second clip then applied at the hinge aspect to lock it in place.

The sliding‐clip technique for renorrhaphy has become widely utilized in open, laparoscopic, and robotic approaches.^8^, ^9^, ^10^ The technique has been adapted, but the fundamental sliding and clipping technique remain common throughout all operative approaches as first described by Agarwal et al.^7^ Today, partial nephrectomy is the recommended surgical approach for T1 renal tumors, particularly those <4 cm (T1a), and is increasingly favored for managing complex renal masses.^11^, ^12^, ^13^ Herein, we describe an Australian experience of partial nephrectomy using the sliding‐clip renorrhaphy technique, from inception to present‐day utilization.

## 2 MATERIALS AND METHODS

### 2.1 Patient selection

After obtaining Institutional Review Board ethical approval, we reviewed a prospectively maintained database of all patients who underwent partial nephrectomy by three experienced urological surgeons (DA, PD, and JK). We included all open, laparoscopic, and robotically assisted partial nephrectomies with the sliding‐clip technique performed between April 2005 and June 2019 at a public and private teaching hospital (Royal Melbourne Hospital and Epworth Hospital).

Patient demographics, perioperative sequelae, and pre‐operative and post‐operative estimated glomerular filtration rate (eGFR) were all recorded. All patients underwent an abdominal CT scan with 3‐mm axial slices to delineate characteristics of tumor location, depth, and proximity to the collecting system. Tumor complexity was defined as low (4‐6 points), intermediate (7‐9 points) or high (10‐12 points), using the RENAL Nephrometry Score.^14^ A thorough chart review was undertaken for each patient to identify 90‐day complications. As Australian patients are both operated on and managed postoperatively in the same health network, most postoperative complications were likely to be identified. Postoperative complications were then graded using the Clavien‐Dindo classification.^15^

### 2.2 Intervention

Across the cohort, the surgical approach varied depending upon tumor size and location. Pre‐operatively, each case was discussed at a multidisciplinary team meeting, where urologists formed a consensus on the indication and strategy for excising the tumor. Tumors located anteriorly and antero‐laterally were approached by transperitoneal laparoscopic approach. Posterior tumors were approached with a retroperitoneal laparoscopic approach. An open approach was used in select patients where laparoscopic approach was considered difficult. Over time, robotically assisted surgeries were used more frequently.

Nonetheless, the principles of surgery were the same in all approaches: first, the hilum and tumor were exposed. The tumor was circumscribed with a safe normal renal parenchymal margin using diathermy, and intraoperative ultrasound was used for all lesions to help identify an adequate excision margin. Next, the renal artery and, in select cases, the renal vein, were clamped using bull dog clamps (at the surgeons’ discretion, patients with superficial tumors generally required clamping of the renal artery only). Tumors were excised with cold scissors and for MIS procedures then placed into a bag.

**FIGURE 1:**
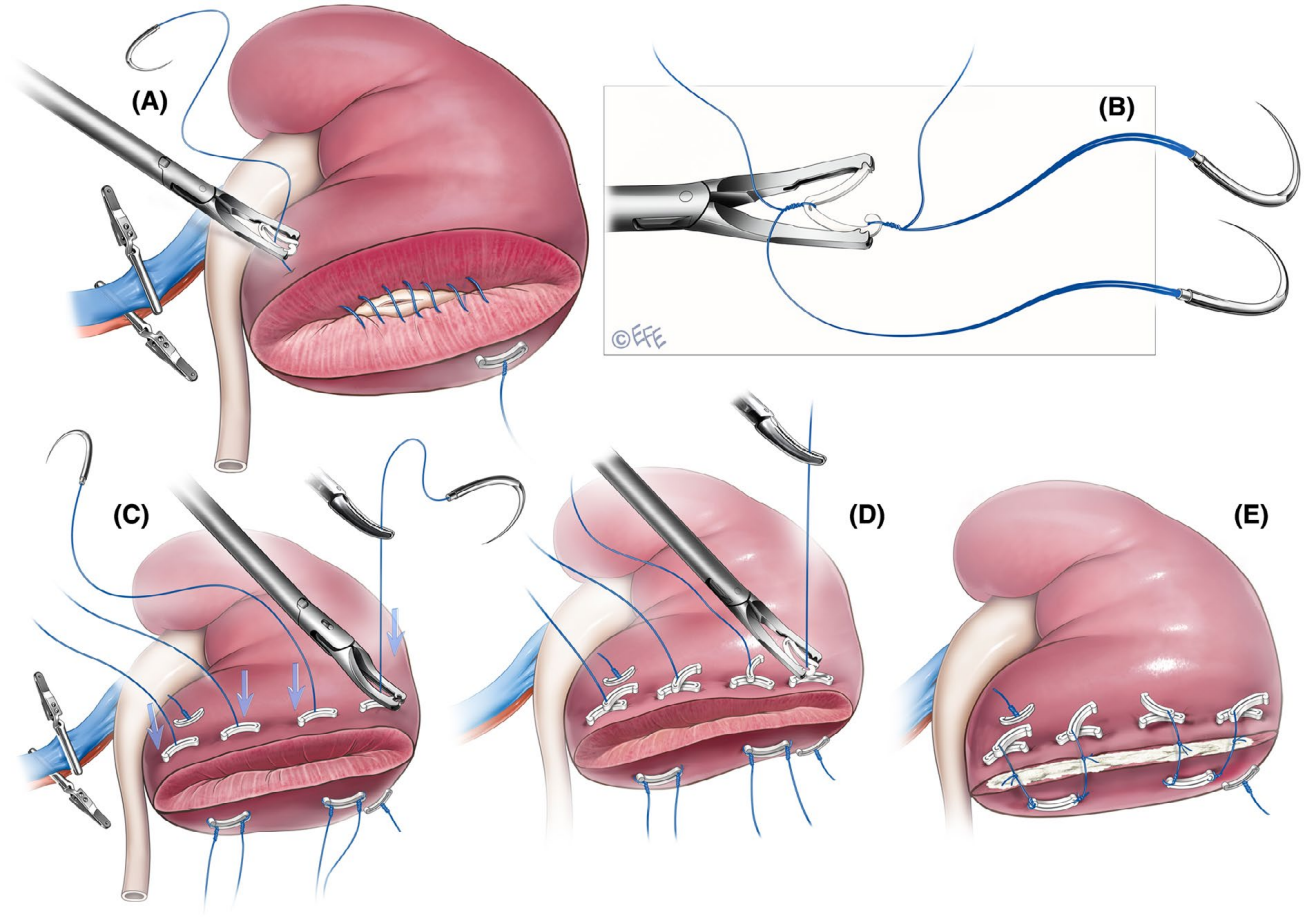
Illustrated steps of the Agarwal Sliding‐Clip Technique. (A) The collecting system and deep layer is closed with a running 3‐0 monocryl (Ethicon Inc., Bridgewater, NJ, USA) or V‐Loc suture (Medtronic, Minneapolis, MN, USA). (B) A double arm suture is prepared with a gold hem‐o‐lok clip (Teleflex Medical, Research Triangle Park, NC, USA) using 0 vicryl suture (Ethicon Inc., Bridgewater, NJ, USA). (C) The superficial layer is closed with 2–3 double arm sutures placed 1 cm apart. Hem‐o‐lok clips are applied and slid down over the vicryl suture to compress the parenchyma achieving hemostasis. Hilar clamps are removed. (D) Additional hem‐o‐lok clips are applied with a locking end to secure the clips (E) If there is any oozing from the wound, the renal defect is filled with loose SURGICEL (Ethicon Inc., Bridgewater, NJ, USA) and Floseal (Baxter Healthcare Corporation, Freemont, CA, USA) and the suture ends are tied across

For all cases included in this sample, the surgeons utilized the sliding‐clip technique (Figure 1A‐E). Renorrhaphy was performed either in single or double layer depending upon the renal defect. The collecting system and deep layer were closed with a running 3‐0 monocryl (Ethicon Inc., Bridgewater, NJ, USA) or V‐Loc (Medtronic, Minneapolis, MN, USA) suture (Figure 1A). The superficial layer was closed with double arm hem‐o‐lok clips as outlined in Figure 1B‐E.

### 2.3 Endpoints and statistical analysis

Patient and disease characteristics were summarized with the median and interquartile range for continuous variables and the frequency and percentage for categorical variables. Baseline patient and tumor characteristics, perioperative parameters, and post‐operative complications were compared across surgical approaches using the Wilcoxon rank‐sum test for continuous variables and the Chi‐squared or Fisher's exact test, as appropriate, for categorical variables. Where multiple tumors were resected, the larger tumor size was used for the analysis.

In this study, the laparoscopic approach was the preferred approach with an open technique being employed for larger or more complex tumors. Later in the series, some laparoscopic cases were undertaken with robotic‐assistance. The data for laparoscopic and robotic cases have been considered both independently and combined as an MIS cohort. Additionally, clinicopathological variables were assessed by whether patients experienced major complications (Clavien‐Dindo ≥ 3), any complication (Clavien‐Dindo ≥ 1), or received a blood transfusion.

A *P*‐value < .05 was considered statistically significant. Statistical analyses were completed using R version 3.5.1 (R Core Development Team, Vienna, Austria).

## 3 RESULTS

Throughout the study period, there were 76 partial nephrectomies, 15 open (including two cases initially performed laparoscopically and converted to an open approach), 48 laparoscopic and 13 robotically assisted operations. The median age at surgery was 56 years (47.5‐64.8). The median pre‐operative eGFR among patients receiving open, laparoscopic, and robotically assisted partial nephrectomies were 74, 83, and > 90 mL/min/1.73 m^2^, respectively, with no significant baseline differences based on procedural approach (Table 1A).

**TABLE 1:** (A) Perioperative characteristics by surgical approach; (B) 90‐day postoperative complications

	Open (n = 15)	Laparoscopic (n = 48)	Robotic (n = 13)	*P*‐value
*(A) Perioperative characteristics*				
Age (median [IQR])	56.00 [45.00, 60.50]	56.00 [50.50, 65.00]	56.00 [50.75, 64.25]	.75
LOS (days) (median [IQR])	6.00 [4.00, 7.50]	3.00 [2.00, 4.00]	4.00 [3.00, 4.00]	<.001
Operation time (median [IQR])	160.00 [102.00, 209.00]	205.00 [156.50, 240.00]	210.00 [200.00, 240.00]	.162
Ischamia time (median [IQR])	0.00 [0.00, 14.25]	21.00 [15.75, 32.00]	20.00 [15.00, 24.00]	<.001
Pre‐op eGFR (median [IQR])	74.00 [53.50, >90.00]	83.00 [71.00, >90.00]	>90.00 [85.00, >90.00]	.277
D1 post‐op eGFR (median [IQR])	50.00 [32.50, 89.00]	65.00 [55.50, >90.00]	83.00 [66.25, >90.00]	.161
Change eGFR (median [IQR])	4.00 [0.00, 29.00]	9.00 [0.00, 17.00]	1.50 [0.00, 12.50]	.706
Transfusion (%)	3 (20.0)	2 (4.2)	2 (15.4)	.076
RENAL nephrometry score (median [IQR])	7.00 [7.00, 8.75]	6.00 [5.00, 7.00]	7.00 [4.00, 9.00]	.242
Surgical complexity (%)				.079
High	2 (20.0)	0 (0.0)	1 (7.7)	
Intermediate	6 (60.0)	10 (40.0)	6 (46.2)	
Low	2 (20.0)	15 (60.0)	6 (46.2)	
Tumor size (mm) (median [IQR])	28.00 [17.50, 33.50]	23.00 [17.00, 30.00]	25.00 [20.00, 46.00]	.174
Pathology (%)				.013
AML	0 (0.0)	2 (4.2)	1 (7.7)	
Chromophobe RCC	3 (20.0)	3 (6.2)	0 (0.0)	
Clear cell RCC	12 (80.0)	35 (72.9)	5 (38.5)	
Oncocytoma	0 (0.0)	0 (0.0)	2 (15.4)	
Papillary RCC	0 (0.0)	5 (10.4)	4 (30.8)	
Unclassified RCC	0 (0.0)	3 (6.2)	1 (7.7)	
T Stage (%) (Malignant tumors)				.003
T1a	11 (73.3)	43 (93.5)	5 (55.6)	
T1b	3 (20.0)	1 (2.2)	4 (44.4)	
T3a	1 (6.7)	2 (4.3)	0 (0.0)	
Positive margin (%)	0 (0)	1 (2.1)	0 (0.0)	1.000
*(B) Postoperative complications*				
Clavien‐Dindo Grade (%)				.009
0	3 (20.0)	33 (68.8)	9 (69.2)	
I	5 (33.3)	8 (16.7)	1 (7.7)	
II	5 (33.3)	5 (10.4)	1 (7.7)	
IIIa	0 (0.0)	1 (2.1)	1 (7.7)	
		Pseudoaneurysm requiring embolization	Iatrogenic pneumothorax. Intercostal catheter inserted on the ward	
IIIb	1 (6.7)	1 (2.1)	1 (7.7)	
	Iatrogenic pneumothorax. Intercostal catheter inserted intraoperatively	Port site hernia, small bowel obstruction	Day 1 Bleed requiring embolization	
IVa	0 (0.0)	0 (0.0)	0 (0.0)	
IVb	1 (6.7)	0 (0.0)	0 (0.0)	
	Bleed requiring surgery & ICU admission for acidosis			
V	0 (0.0)	0 (0.0)	0 (0.0)	
Any‐Clavien‐Dindo grade (%)	12 (80.0)	15 (31.2)	4 (30.8)	.003
Clavien‐Dindo grade ≥3 (%)	2 (13.3)	2 (4.2)	2 (15.4)	.188

Abbreviations: D1, day one; LOS, length of stay.

The median tumor size was 25 mm (17.5‐32.0). The median tumor size in the open partial nephrectomy group was larger than the MIS groups; however, this difference was not statistically significant. Most tumors were staged as pT1a (≤4 cm), 73% and 87% for open and MIS, respectively. When subdividing MIS into laparoscopic and robotically assisted approaches, the robotically assisted surgeries had a higher proportion of T1b [4‐7 cm] tumors (44% vs 2%; *P* = .003), perhaps reflecting contemporary case selection patterns for robotically assisted partial nephrectomies. Indeed, with superior vision and ergonomics, partial nephrectomy for complex lesions may be more feasible using robotically assisted approaches than laparoscopy. Final pathology returned 66% clear cell RCC, while 7% of tumors were benign. Only one MIS case had a positive malignant margin. Two patients had bilateral tumors simultaneously excised: Patient 1 had a 50 mm and a 54 mm tumor removed, both with clear cell RCC T1b pathology, and Patient 2 had a 30 mm and a 12 mm tumor removed, both with clear cell RCC T1a pathology; both surgeries had negative margins.

Surgical complexity was available for 63% of cases, of which only 20% of open partial nephrectomies had low surgical complexity. Conversely, 60% of laparoscopic and 46% of robotically assisted surgeries had low surgical complexity. This difference in surgical complexity between open surgery and MIS was statistically significant (*P* = .034). Ischemia time varied significantly by approach with 10 open partial nephrectomies performed off‐clamp and four employing cold ischemia. Between laparoscopic and robotically assisted surgeries, median clamp time was comparable at 21 minutes and 20 minutes, respectively. Total procedure time for open surgeries was less than MIS, although this was not statistically significant (*P* = .064).

Post‐operatively, 31 (69%) patients experienced a complication within 90 days of surgery (predominantly Clavien‐Dindo grade I events). Only six (8%) patients had major post‐operative complications (Clavien‐Dindo grade ≥ 3). Interestingly, while patients undergoing open procedures were more likely to experience a complication (*P* = .003), there was no difference in major complication rates between open, laparoscopic, and robotically assisted approaches (Table 1B). Seven patients (9%) received a perioperative blood transfusion, of these only two patients had greater than two units of packed red blood cells transfused. Those two cases involved sub‐segmental renal artery embolization, one of whom proceeded to theater for hemostasis. Laparoscopic surgery had a lower overall rate of transfusions (4%), although this was not statistically significant. Change in day one post‐operative eGFR from baseline level did not vary by approach. Finally, MIS had a significantly shorter median length of inpatient stay: 3 days vs 6 days for open surgery (*P* < .001).

**FIGURE 2:**
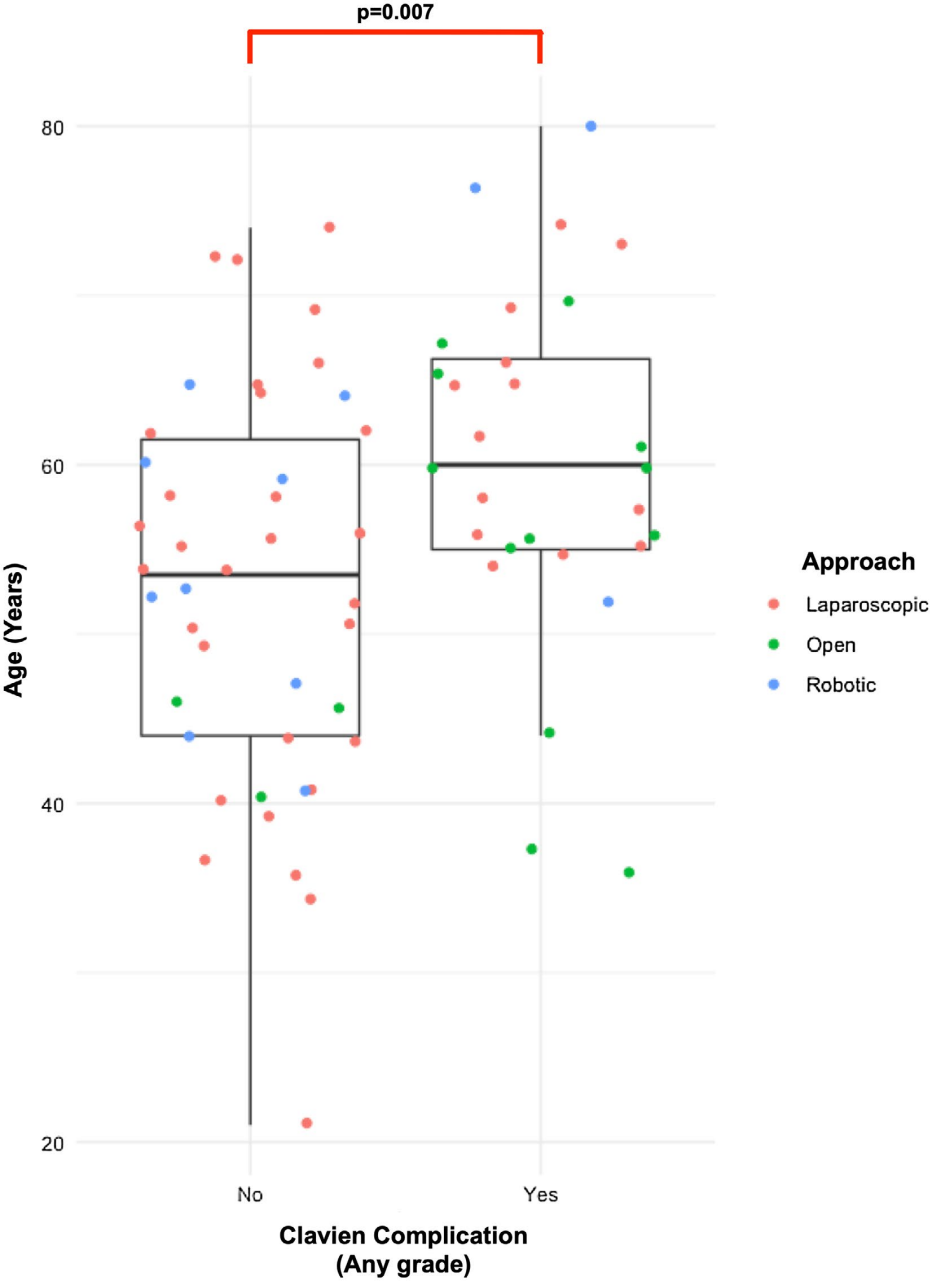
Features associated with any‐grade complications. Age is measured at the time of surgery. Any Clavien‐Dindo complication (Grade ≥ 1) within 90 days of surgery is included

The only independent covariates associated with any‐Clavien‐Dindo grade complications were older patients (*P* = .007), open surgery (*P* = .003), and longer total length of stay (*P* < .001) (Figure 2). Blood transfusions were associated with older patients (*P* = .033) and lower day one post‐operative eGFR (*P* = .043), likely reflecting intraoperative volume depletion. Major complications were associated with a higher median RENAL Nephrometry Score (9 vs 6; *P* = .016) and, among the malignant tumors, major complications were also associated with a higher proportion of pT stage > T1a tumors (50% vs 13%; *P* = .045), also likely reflecting the greater intraoperative complexity.

## 4 DISCUSSION

Our case series encompasses the initial learning curve through to present‐day routine standard of practice using the sliding‐clip technique for renorrhaphy partial nephrectomy. The advantages of the sliding‐clip technique are that it minimizes the risk of parenchymal tearing by sutures, provides effective hemostasis, and decreases warm ischemia time by the virtue of its knotless technique.^7^, ^8^, ^16^, ^17^ Herein, we report that the sliding‐clip technique has low complication rates, acceptable operative time and preserves renal function among open, and MIS approaches.

Presently, partial nephrectomy is the gold standard for excising pT1a renal tumors and increasingly larger, more complex renal masses.^13^ Aside from the debate about oncological equivalency to radical nephrectomy, the main concern previously restricting the uptake of partial nephrectomy was morbidity associated with achieving hemostasis and urinary leaks. The sliding‐clip renorrhaphy technique and its variations have assisted in improving safety and outcomes therein. Despite including the first‐in‐human cases, we report low overall rates of transfusions when utilizing the sliding‐clip. Only seven patients had blood transfusions in our series, with two (3%) receiving more than two units of packed red blood cells. Overall, the low transfusion requirements indicate that excellent intraoperative hemostasis was achieved.

A systematic review by Bortolo et al outlines the evolution of renorrhaphy techniques for MIS and their effect on perioperative outcomes.^11^ The review found that prior to 2007, conventional methods for parenchymal closure in partial nephrectomy relied on the use of surgical knots,^18^, ^19^, ^20^ with some techniques using a bolster and surgical knots^19^, ^21^, ^22^ while others included hemostatic agents alone or in combination with sutures.^20^, ^23^ In 2004, Orvieto et al described a knotless technique for renorrhaphy utilizing LapraTy clips instead of surgical knots.^22^, ^24^ Knotless techniques such as this were utilized in several studies of laparoscopic partial nephrectomy thereafter.^17^, ^25^ None of these techniques described using a sliding‐clip approach for fixation, until Agarwal et al published an illustrated description of a knotless, sliding‐clip technique for renorrhaphy in 2007 utilizing Hem‐o‐lok® clips.^7^ In 2008, Bhayani et al described and subsequently published the sliding technique for robotic surgery, thereby advancing the technique into the MIS setting.^8^

As the technique of sliding‐clip renorrhaphy transitioned from laparoscopic to robotically‐ assisted surgery, it further evolved to be utilized in more complex, higher stage tumors. Although major post‐operative complications were significantly associated with tumor stage and complexity, the small number of these cases may reflect the initial learning curve. Additional cases are required to better understand this association. However, complications after partial nephrectomy for complex tumors may also decline with ongoing advancements in surgical techniques. Although the principles of sliding‐clip renorrhaphy continue to form the basis of standard renorrhaphy technique today in open, laparoscopic, and robotic partial nephrectomy, subtle modifications have occurred. Specific variations include the use of running sutures, a single suture line, and self‐retaining barbed sutures avoiding the need to retighten, all of which have been associated with reductions in operative and ischemia times.^11^ Other modifications include using a bolster or non‐bolstered horizontal mattress closure of the capsule^26^ or even omitting to close the cortex.^27^ A trial randomising patients to double‐layer or medullary renorrhaphy alone is currently accruing with preliminary results potentially suggesting that the closure of the deep‐layer alone may be superior.^28^ Furthermore, these variations may also impact longer term total renal volume loss.^29^ Incorporation of these elements when undertaking sliding‐clip renorrhaphy may further improve the safety and utility of partial nephrectomy for managing renal masses. Nonetheless, each of these adaptions retains the central sliding and clipping component.

The results of our study highlight that the sliding‐clip technique for renorrhaphy provides effective collecting system closure and hemostasis during open and laparoscopic partial nephrectomy. While open surgery compared with MIS was associated with longer admissions and more complications, there was no increase in the rate of major complications. Differences between approaches may be influenced by the multidisciplinary team typically advocating for open surgery for solitary kidneys and more complex cases; additionally, the longer length of stay for open procedures may allow for more accurate detection of minor complications. Only one case returned to theater for post‐operative bleeding and there were no collecting system leaks in this study. These results are similar to those of other larger studies^16^, ^17^, ^26^, ^30^ and in this case, the use of a double arm Hem‐o‐lok® may have contributed to achieving effective hemostasis and collecting system closure. Increased utilization of robotically assisted partial nephrectomy may further reduce the need for transfusions. Uniquely, our results capture the initial learning curves of the sliding‐clip technique first with laparoscopic and open surgeries and later with a robotically assisted approach, together indicating that the sliding‐clip technique can be learned quickly and safely.

Our study comprises a relatively small number of cases stretching over a decade. While this may under‐power some statistical tests, the absolute numbers also reflect real‐world surgical patterns within a health system without centralized specialization. Indeed, the hospitals in this study are two of the state's largest and include one of the only three public hospitals in the state with infrastructure for robotically assisted surgery. The safety and efficacy of the sliding clip renorrhaphy have been reported in other series and this technique is now widely used in several larger centers. As larger studies have compared the sliding‐clip technique with other renorrhaphy techniques, we instead provide a historical context for the development of the sliding‐clip technique and the patient outcomes therein. Finally, our study supports the contention that relatively lower volume institutions can innovate by repurposing existing medical devices to improve global standards of practice. Foreseeably, this technique will continue to evolve with iterative technological refinements by urologists further improving outcomes.^31^

## 5 CONCLUSION

After 150 years of incremental improvement, the partial nephrectomy for T1 renal tumors has emerged as the gold standard treatment. The widespread adoption of nephron‐sparing surgery is predominantly due to reduced perioperative morbidity and preserved long‐term renal function. While larger partial nephrectomy studies in the literature describe how the sliding‐clip technique for renorrhaphy facilitates effective hemostasis and shortens ischemia time, this paper serves to provide a historical perspective by describing the first‐in‐human experience. Furthermore, the study demonstrates how the technique, which is steadily being promulgated within the urology community, was safely applied in lower volume teaching hospitals. Finally, this experience demonstrates that ingenuity from a single institution can lead to changes in practices worldwide.

## DISCLOSURE STATEMENT

The authors have no relevant disclosures pertaining to this manuscript.

## References

[1] Simon G . Chirurgie der Nieren. vol II. Stuttgart: Ferdinand Enke; 1876.

[2] Herr HW . A history of partial nephrectomy for renal tumors. J Urol. 2005;173:705–8. 15711247 10.1097/01.ju.0000146270.65101.1d

[3] Luciani LG , Cestari R , Tallarigo C . Incidental renal cell carcinoma‐age and stage characterization and clinical implications: study of 1092 patients (1982–1997). Urology. 2000;56:58–62. 10869624 10.1016/s0090-4295(00)00534-3

[4] Van Poppel H , Da Pozzo L , Albrecht W , Matveev V , Bono A , Borkowski A , et al. A prospective, randomised EORTC intergroup phase 3 study comparing the oncologic outcome of elective nephron‐sparing surgery and radical nephrectomy for low‐stage renal cell carcinoma. Eur Urol. 2011;59:543–52. 21186077 10.1016/j.eururo.2010.12.013

[5] Scosyrev E , Messing EM , Sylvester R , Campbell S , Van Poppel H . Renal function after nephron‐sparing surgery versus radical nephrectomy: results from EORTC randomized trial 30904. Eur Urol. 2014;65:372–7. 23850254 10.1016/j.eururo.2013.06.044

[6] Van Poppel H , Da Pozzo L , Albrecht W , Matveev V , Bono A , Borkowski A , et al. A prospective randomized EORTC intergroup phase 3 study comparing the complications of elective nephron‐sparing surgery and radical nephrectomy for low‐stage renal cell carcinoma. Eur Urol. 2007;51:1606–15. 17140723 10.1016/j.eururo.2006.11.013

[7] Agarwal D , O’Malley P , Clarke D , Rao R . Modified technique of renal defect closure following laparoscopic partial nephrectomy. BJU Int. 2007;100:967–70. 17822478 10.1111/j.1464-410X.2007.07104.x

[8] Bhayani SB , Figenshau RS . The Washington University Renorrhaphy for robotic partial nephrectomy: a detailed description of the technique displayed at the 2008 World Robotic Urologic Symposium. J Robot Surg. 2008;2(3):139–40. 10.1007/s11701-008-0096-4 27628249

[9] Masumori N , Itoh N , Takahashi S , Kitamura H , Nishida S , Tsukamoto T . New technique with combination of felt, Hem‐o‐lok and Lapra‐Ty for suturing the renal parenchyma in laparoscopic partial nephrectomy. Int J Urol. 2012;19:273–6. 22168748 10.1111/j.1442-2042.2011.02935.x

[10] Wahafu W , Ma X , Li HZ , Ding Q , Wang BJ , Shi TP , et al. Evolving renorrhaphy technique for retroperitoneal laparoscopic partial nephrectomy: single‐surgeon series. Int J Urol. 2014;21(9):865–73. 10.1111/iju.12470 24780100

[11] Bertolo R , Campi R , Klatte T , Kriegmair MC , Mir MC , Ouzaid I , et al. Suture techniques during laparoscopic and robot‐assisted partial nephrectomy: a systematic review and quantitative synthesis of peri‐operative outcomes. BJU Int. 2019;123(6):923–46. 10.1111/bju.14537 30216617

[12] Campbell S , Uzzo RG , Allaf ME , Bass EB , Cadeddu JA , Chang A , et al. Renal mass and localized renal cancer: AUA guideline. J Urol. 2017;198(3):520–9. 10.1016/j.juro.2017.04.100 28479239

[13] Ljungberg B , Albiges L , Abu‐Ghanem Y , Bensalah K , Dabestani S , Fernández‐Pello S , et al. European Association of Urology guidelines on renal cell carcinoma: the 2019 update. Eur Urol. 2019;75(5):799–810. 10.1016/j.eururo.2019.02.011 30803729

[14] Kutikov A , Uzzo RG . The R.E.N.A.L. nephrometry score: a comprehensive standardized system for quantitating renal tumor size, location and depth. J Urol. 2009;182:844–53. 19616235 10.1016/j.juro.2009.05.035

[15] Dindo D , Demartines N , Clavien PA . Classification of surgical complications: a new proposal with evaluation in a cohort of 6336 patients and results of a survey. Ann Surg. 2004;240:205–13. 15273542 10.1097/01.sla.0000133083.54934.ae PMC1360123

[16] Benway BM , Wang AJ , Cabello JM , Bhayani SB . Robotic partial nephrectomy with sliding‐clip renorrhaphy: technique and outcomes. Europ Urol. 2009;55(3):592–9. 10.1016/j.eururo.2008.12.028 19144457

[17] Canales BK , Lynch AC , Fernandes E , Anderson JK , Ramani AP . Novel Technique of Knotless Hemostatic Renal Parenchymal Suture Repair During Laparoscopic Partial Nephrectomy. Urology. 2007;70(2):358–9. 10.1016/j.urology.2007.04.031 17826508

[18] Gill IS , Desai MM , Kaouk JH , Meraney AM , Murphy DP , Sung GT , et al. Laparoscopic partial nephrectomy for renal tumor. J Urol. 2002;167:469–76. 10.1097/00005392-200202000-00005 11792899

[19] Desai MM , Gill IS , Kaouk JH , Matin SF , Novick AC . Laparoscopic partial nephrectomy with suture repair of the pelvicaliceal system. Urology. 2003;61:99–104. 12559277 10.1016/s0090-4295(02)02012-5

[20] Porpiglia F , Renard J , Billia M , Morra I , Terrone C , Scarpa RM . Biological glues and collagen fleece for hemostasis during laparoscopic partial nephrectomy technique and results of prospective. Study. 2007;21(4):423–8. 10.1089/end.2006.0265 17451336

[21] Heinrich E , Egner T , Noe M , Schiefelbein F , Schoen G . Organ‐Preserving Endoscopic Kidney Cancer Resection. Eur Urol. 2006;50(4):732–7. 10.1016/j.eururo.2006.07.040 16930815

[22] Orvieto MA , Chien GW , Tolhurst SR , Rapp DE , Steinberg GD , Mikhail AA , et al. Simplifying laparoscopic partial nephrectomy: Technical considerations for reproducible outcomes. Urology. 2005;66(5):976–80. 10.1016/j.urology.2005.05.013 16286106

[23] Wille AH , Tüllmann M , Roigas J , Loening SA , Deger S . Laparoscopic partial nephrectomy in renal cell cancer—Results and reproducibility by different surgeons in a High Volume Laparoscopic Center. Eur Urol. 2006;49(2):337–43. 10.1016/j.eururo.2005.11.016 16413957

[24] Orvieto MA , Chien GW , Laven B , Rapp DE , Sokoloff MH , Shalhav AL . Eliminating knot tying during warm ischemia time for laparoscopic partial nephrectomy. J Urol. 2004;172:2292–5. 15538251 10.1097/01.ju.0000145535.48499.c1

[25] Abukora F , Nambirajan T , Albqami N , Leeb K , Jeschke S , Gschwendtner M , et al. Laparoscopic Nephron Sparing Surgery: Evolution in a Decade. Eur Urol. 2005;47(4):488–93. 10.1016/j.eururo.2004.12.021 15774247

[26] Kaouk JH , Hillyer SP , Autorino R , Haber GP , Gao T , Altunrende F , et al. 252 robotic partial nephrectomies: evolving renorrhaphy technique and surgical outcomes at a single institution. Urology. 2011;78(6):1338–44. 10.1016/j.urology.2011.08.007 22001098

[27] Bahler CD , Dube HT , Flynn KJ , Garg S , Monn MF , Gutwein LG , et al. Feasibility of omitting cortical renorrhaphy during robot‐assisted partial nephrectomy: a matched analysis. J Endourol. 2015;29(5):548–55. 10.1089/end.2014.0763 25616087

[28] Fenner A , Bahler C . Investigating a novel modifiable factor affecting renal function after partial nephrectomy: cortical renorrhaphy. Proceedings of IMPRS. 2019;2(1). 10.18060/23462

[29] Plattner HS , Sundaram CP , Cheng L , Bahler CD . Renal volume loss during partial nephrectomy due to resected healthy parenchyma: a tool for quick estimation. J Endourol. 2020;34(8):856–61. 10.1089/end.2020.0314 32336144

[30] Crestani A , Giannarini G , Rossanese M , Calandriello M , Palumbo V , Valotto C , et al. Sliding‐clip technique for renorrhaphy improves perioperative outcomes of open partial nephrectomy. Scand J Urol. 2018;52(5‐6):401–6. 10.1080/21681805.2018.1513066 30336721

[31] Lawrentschuk N . Urology trial success—get urologists involved early. BJU Int. 2019;124:4. 10.1111/bju.14933 31746142

